# Effectiveness of online education on thermography-based diabetic foot ulcer prevention for wound care specialists: a single-group quasi-experimental study

**DOI:** 10.1007/s13340-025-00791-4

**Published:** 2025-01-31

**Authors:** Muhammad Aminuddin, Sukarni, Haryanto, Suriadi Jais, Amika Yamada, Kanae Mukai, Makoto Oe

**Affiliations:** 1 Division of Health Sciences, Graduate School of Medical Sciences, Kanazawa University, 5-11-80, Kodatsuno, Kanazawa, Ishikawa Japan; 2 Nursing Department, Faculty of Medicine, Mulawarman University, Jl. Anggur No. 88, Samarinda, Kalimantan Timur Indonesia; 3 Nursing Department, Faculty of Medicine, Tanjungpura University, Jl. Prof. H. Hadari Nawawi, Pontianak, Kalimantan Barat Indonesia; 4 Institut Teknologi Dan Kesehatan Muhamamdiyah Kalimantan Barat, Jl. Sungai Raya Dalam II, Kubu Raya, Kalimantan Barat Indonesia; 5 Faculty of Health Sciences, Institute of Medical, Pharmaceutical and Health Sciences, Kanazawa University, 5-11-80, Kodatsuno, Kanazawa, Ishikawa Japan

**Keywords:** Diabetic feet, Knowledge, E-learning, Infrared

## Abstract

**Background:**

While there is an urgent need worldwide to establish methods that prevent diabetic foot ulcers, the effectiveness of a prevention protocol using thermography has been reported. As the number of diabetic patients in Indonesia is increasing, an online program for wound care specialists was developed to disseminate this protocol. The present study evaluated the impact of an online program on wound care specialists' knowledge of diabetic foot ulcer prevention using thermography.

**Methods:**

This single-group quasi-experimental study was conducted in cooperation with the Indonesian Wound Enterostomal Continence Nurses Association with regard to curriculum and content design, and the evaluation methods of online education for thermography-based diabetic foot ulcer prevention using the learning management system. A questionnaire with 50 multiple-choice questions previously validated for content and readability on the knowledge of diabetic foot ulcer prevention using thermography was used for training evaluations.

**Results:**

Of 106 Indonesian wound care specialists evaluated, the paired t-test revealed a significant difference between the before and after training questionnaire scores on knowledge (52.0 ± 10.3, 85.2 ± 10.6, respectively, *p* < 0.001). Repeated measures analysis of variance revealed interactions between time (before and after training) and gender, and between time and type of certificate (*p* = 0.046, *p* = 0.014, respectively).

**Conclusions:**

An asynchronous e-learning program is an effective method to increase wound care specialists' knowledge of diabetic foot ulcer prevention. These findings suggest that online educational interventions are effective and can be tailored to meet the needs of healthcare professionals, thereby ultimately contributing to better patient care outcomes in preventing DFUs.

## Background

Diabetic foot ulcers (DFUs) are one of the complications of diabetes, with their low healing rate and infections affecting physical prognosis, life prognosis, and patient quality of life, along with being a burden on healthcare resources [1–3]. The annual incidence of DFUs is estimated to be 2–2.2% [4], with a lifetime incidence of 19–34%. Furthermore, the recurrence rate is also high, with these ulcers found again in 40% within a year and 60% within 3 years [5]. With the rapid increase in the number of patients with diabetes, establishing measures to prevent DFUs is an urgent worldwide issue.

One of the recommended effective DFU prevention methods is the measurement of foot temperatures. When performing DFU prevention protocols using thermography, this can reduce the incidence of ulcers by 0.2% in the first 12 months [6]. Thermographic images of the foot allow patients and healthcare professionals to share the early stages of DFUs by visually detecting invisible signs of inflammation as an increase in the skin temperature [6, 7]. When the thermography DFU prevention protocols observe an elevated skin temperature, the healthcare provider then instructs the patient to more frequently see the physician. Furthermore, this also helps provide patients with motivation for self-care related to foot management. Care using visualization techniques such as thermography can also contribute to helping to prevent the development of DFUs.

In Indonesia, where there has been a rapid increase in the number of patients with diabetes, thermographic foot care has been reported to be effective in preventing recurrent DFUs [8]. In a previous study, two wound care specialists underwent 10 days of training in English at a Japanese hospital that introduced DFU prevention protocols using thermography. Wound care specialists play a central role in DFU prevention in Indonesia, which does not have a system of Certified Diabetes Educator similar to that found in Japan. Thus, in order to disseminate DFU prevention protocols using thermography in Indonesia in the future, not only for recurrence but also for occurrence, it will be necessary for wound care specialists to acquire the latest knowledge of DFU preventive care using thermography and patient education skills in the Indonesian language. In addition, as discovered during the COVID-19 pandemic, it is important that these types of training programs be made available online [9]. Online training using e-modules has been shown to significantly improve nurses' knowledge about diabetes and diabetic foot care [10, 11]. In contrast to previous research, the online training in this study used videos related to the prevention of DFUs. Therefore, in this study, an online program on DFU prevention protocols using thermography was created in the Indonesian language, with the aim of the study to examine changes in the knowledge of wound care specialists. Proving the effectiveness of this online training program on providing knowledge will be a prerequisite for future validation of the effectiveness of DFU preventive care using thermography when graduates of the program begin to provide care to their own patients.

## Methods

### Study design

This study was a single-group quasi-experimental design that compared the knowledge of the non-randomization group of wound care specialists regarding DFU preventive care using thermography.

### Sampling method and participants

The population of this study was wound care specialists. The participants were recruited in collaboration with the Indonesian Wound Enterostomal Continence Nurses Association (InWECNA), which has 25 representative branches throughout Indonesia. In this study, 19 InWECNA branches from 19 participating provinces and six islands sent a total of 150 prospective participants for enrollment in the course. The sample size was calculated using G*Power software. Based on published data from previous studies, the average increase in diabetic foot care knowledge score after online training was 40% [10]. Therefore, we assumed an effect size of 0.4. With a significance power of 95% and a type 1 error rate of 0.05 in a paired t-test, the required sample size was 84. Given the high dropout rate previously established, we set the sample size to 120, allowing for a dropout rate of 30%. The inclusion criteria were being a wound care nurse specialist, experience working in wound care, and the willingness to be a participant.

### Data collection

Data collection used a structured questionnaire. The socio-demographic data and knowledge scores before and after the training were collected using the questionnaire that was contained in the learning management system (LMS). All questionnaires were pre-tested by a wound care specialist to ensure the clarity of the questions and avoid ambiguity. We investigated the participants' demographics, including age, gender, educational level, work experience, and certificate type. Knowledge scores regarding DFU prevention were collected before and after the training. In addition, this study used online surveys to evaluate the level of satisfaction of the participants.

### Steps of the online training program performed

Step 1: Develop an online training program for thermography-based DFU prevention.

This program was prepared by the person in charge based on the results of visits to the diabetic foot care outpatient clinic of the University of Tokyo Hospital and the SCHUH-BELL Footcare Center Japan. These facilities have both implemented the thermography-based DFU prevention treatment protocol. Next, the e-learning program curriculum was then developed based on the visit results and the thermography-based DFU prevention treatment protocol. The curriculum was created in the Indonesian language, and then prepared and validated by Indonesian wound care experts. The online training program consists of videos using a LMS and 17 topics (Table 1). The video topics consist of basic knowledge of diabetic foot ulcer (first video), preventive care for diabetic foot ulcers using visualization technology (second video), self-efficacy (third video), algorithm for prevention of diabetic foot ulcer (fourth video), standard care of monofilament evaluation (fifth video), standard care of ABI evaluation (sixth video), standard care of macroscopic observation (seventh video), standard care of plantar pressure evaluation (eighth video), standard care of inflammation evaluation (ninth video), standard care and education of LOPS (tenth video), standard care and education of PAD (eleventh video), standard care and education of suspected tinea infection (twelfth video), standard care and education of dry skin, fissure (thirteenth video), standard care and education of callus (fourteenth video), standard care and education of tinea infection (fifteenth video), standard care and education of plantar pressure evaluation with no increased pressure (sixteenth video), and case study (seventeenth video). The topics and contents of the video have also been tested and edited by Indonesian wound care specialists who manage the InWECNA. In addition, two wound care nurse specialists from hospitals and private clinic practices were involved in providing input related to the readability test of the video content and the use of LMS. The training program was carried out using the asynchronous e-learning program. This method allows participants to take part in training at both different times and places in accordance with the participants' time availability.

**Table 1:** Training topics

Training topics of thermography-based foot care on the prevention of diabetic foot ulcers
1. Basic knowledge of diabetic foot ulcer
2. Preventive care for diabetic foot ulcers using visualization technology
3. Self-efficacy
4. Algorithm for prevention of diabetic foot ulcer
5. Standard care of monofilament evaluation
6. Standard care of ABI evaluation
7. Standard care of macroscopic observation
8. Standard care of plantar pressure evaluation
9. Standard care of inflammation evaluation
10. Standard care and education of LOPS
11. Standard care and education of PAD
12. Standard care and education of suspected tinea infection
13. Standard care and education of dry skin, fissure
14. Standard care and education of callus
15. Standard care and education of tinea infection
16. Standard care and education of plantar pressure evaluation with no increased pressure
17. Case study

*ABI* ankle-brachial index, *LOPS* loss of protective sensation, *PAD* peripheral artery disease

Step 2: Development of the multiple choice questionnaire (MCQ) to test the current knowledge of thermography-based DFU prevention.

An MCQ was developed to assess the participants' knowledge of thermography-based DFU prevention. The questionnaire consisted of 50 questions (Table 2) that were created based on the training curriculum. Fifty questions were arranged based on each learning video. Multiple-choice questions consist of questions numbered 1–5 (first video), 6– 8 (second video), 9– 11 (third video), 12–15 (fourth video), 16–19 (fifth video), 20–23 (sixth video), 24– 27 (seventh video), 28–31 (eighth video), 32–35 (ninth video), 36 (tenth video), 37 (eleventh video), 38–39 (twelfth video), 40–41 (thirteenth video), 42–44 (fourteenth video), 45–46 (fifteenth video), 47–48 (sixteenth video) and 49–50 (seventeenth video). There are three possible answer options for each statement: true, false, and do not know. The correct answer is given a score of 2, while the wrong and unknown answer is given a score of 0, with possible totals of a lowest score of 0 and a highest score of 100. The questionnaire was tested for face validity and content validity by a panel of wound care specialist experts at the InWECNA. Wound care specialists in Indonesia edited the questions, while two specialists were asked to answer the questions to assess the difficulty in answering the material. After the questionnaire was finalized, the internal consistency reliability was measured using Cronbach's alpha. 40 wound care specialists answered a final questionnaire before the training began, and Cronbach's alpha was 0.87, calculated.

**Table 2:** Multiple choice questionnaire on knowledge of thermography-based diabetic foot ulcer prevention and correct answer rate (*n* = 106)

Questionnaire items	Correct answer rate (%)	
	Before training	After training
Basic knowledge of diabetic foot ulcer	38.1	91.5
1. Based on the International Working Group on the Diabetic Foot (IWGDF), one of the outcomes of diabetes-related foot diseases is amputation. (True)	45.3	100
2. Ischemia is the main factor causing diabetic foot ulcers. (False)	13.2	88.7
3. Callus formation can cause bleeding in the skin. (True)	32.1	90.6
4. Peripheral ischemia is a trigger for foot deformity. (False)	35.9	88.7
5. Diabetic neuropathic ulcers are more painful than diabetic ischemic ulcers. (False)	64.2	89.6
Preventive care for diabetic foot ulcers using visualization technology	39.3	88.1
6. Screening of diabetics at very low risk of foot ulceration (IWGDF risk 0) is performed every six months. (False)	25.5	87.7
7. Thermography is a technology that can diagnose ischemic diabetic foot ulcers. (False)	81.1	90.6
8. Areas with decreased skin temperature are an indicator of a pre-ulcerative condition in the legs. (False)	11.3	85.9
Self-efficacy	58.8	90.6
9. Foot care to prevent diabetic foot ulcers must be carried out by health workers. (False)	49.1	88.7
10. If the patient makes a mistake, direct the patient to think about where they went wrong. (True)	74.5	92.5
11. The appearance of other people does not influence the development of a person's self-efficacy. (False)	52.8	90.6
Algorithm for prevention of diabetic foot ulcer	50.5	89.9
12. Patients with LOPS are advised to have daily foot examinations. (True)	83.0	94.3
13. If the result of the PAD is positive, it is recommended to carry out an annual check-up. (False)	33.0	84.0
14. Based on the DFU prevention algorithm, if an increase in skin temperature is found outside the non-ulcerated ulcer area, it is recommended to have a monthly check-up. (False)	18.9	85.9
15. In the case of an increase in skin temperature in the non-ulcerated wound area, the patient must carry out routine monthly check-ups. (True)	67.0	95.3
Standard care of monofilament evaluation	40.6	81.1
16. The duration of the monofilament inspection is about 2 s from the time the monofilament is attached until the monofilament is removed. (True)	76.4	85.9
17. In the monofilament examination, the filament can be attached to a healed scar. (False)	35.9	85.9
18. The protective sensation is said to be lost if the patient does not feel at least one of the three examinations at one site. (False)	16.0	79.3
19. Monofilament can be used for a maximum of 70–90 patients for 24 h. (False)	33.9	73.6
Standard care of ABI evaluation	52.2	79.2
20. ABI examination can be carried out with the patient sitting. (False)	71.7	79.3
21. To calculate the ABI value, we divide the upper arm systolic blood pressure by the ankle systolic blood pressure. (False)	36.8	82.1
22. In the calculation of ABI, if the systolic pressure of the upper arm differs between the right and left, then the one used is the lowest. (False)	37.8	74.5
23. ABI value < 0.9 is considered a sign of normal blood flow. (False)	62.3	81.1
Standard care of macroscopic observation	80.4	87.3
24. The purpose of using a background during photography is to make it easier to see the position of the feet. (True)	77.4	84.0
25. The images are taken sequentially to record them consistently. (True)	91.5	93.4
26. If non-ulcerated lesions are found to occur, then ask about the process of development. (True)	87.7	88.7
27. The purpose of the macroscopic observation examination is to note non-ulcerated wounds. (True)	65.1	83.0
Standard care of plantar pressure evaluation	71.7	87.7
28. The footprint is an examination to evaluate plantar pressure. (True)	84.0	94.3
29. Footprint examination can be performed in a sitting position. (False)	52.8	84.9
30. A portion where the ink is darkly transferred shows high pressure. (True)	82.1	88.7
31. On the results of the footprint examination, if the high pressure is not present in the callus area, it may be caused by shoes that do not fit. (True)	67.9	83.0
Standard care of inflammation evaluation	41.7	79.2
32. When taking thermographic images, the patient's knees should be placed close to each other. (False)	30.2	83.0
33. On a thermography examination, an increase in skin temperature extending towards the arch of the foot can be a sign of pre-ulcerative pathology. (True)	74.5	77.4
34. The results of a thermography examination where there is an increase in skin temperature that extends to the ankle area are a sign of cellulitis. (False)	9.4	76.4
35. In patients with ischemia, an increase in skin temperature may not be visible. (True)	52.8	80.2
Standard care and education of LOPS	74.5	90.6
36. The recommended behavior change for LOPS sufferers is washing their feet every day. (True)	74.5	90.6
Standard care and education of PAD	34.9	88.7
37. Patients with PAD are advised to walk in socks without shoes while at home to prevent trauma. (False)	34.9	88.7
Standard care and education of suspected tinea infection	72.2	82.5
38. The nurse removed the infected nail (tinea) because it was very brittle. (True)	57.5	81.1
39. One sign of suspected tinea infection is the thickening of the nails. (True)	86.8	84.0
Standard care and education of dry skin, fissure	45.3	87.7
40. A sign of dry skin is the presence of white spots on the skin. (True)	60.4	91.5
41. Moisturizer is applied to the interdigital cleft of the feet with dry skin. (False)	30.2	84.0
Standard care and education of callus	58.2	83.0
42. An increase in foot temperature indicates a risk of callus. (False)	50.0	80.2
43. After smoothing the callus with a file, apply moisturizing cream to the callus. (True)	91.5	92.5
44. Footwear that is not cramped can reduce the development of calluses. (False)	33.0	76.4
Standard care and education of tinea infection	47.7	80.7
45. In the case of tinea unguium, use liquid antifungal drugs applied to the base of the nail. (True)	74.5	77.4
46. In the case of tinea pedis, antifungal ointment is applied only to the affected area. (False)	20.8	84.0
Standard care and education of plantar pressure evaluation with no increased pressure	84.0	86.8
47. If the footprint results show no increase in pressure in the callus area, then the load while walking is the cause of callus formation. (True)	73.6	78.3
48. Gait can affect the formation of calluses on the soles of the feet. (True)	94.3	95.3
Case study	26.5	74.1
49. Monofilament test should be performed before the thermography examination. (False)	12.3	70.8
50. ABI measurements should be carried out after thermography examination. (True)	40.6	77.4

*ABI* ankle-brachial index, *LOPS* loss of protective sensation, *PAD* peripheral artery disease

Step 3: Creating a learning program on thermography-based DFU prevention.

Prospective participants who were submitted by InWECNA and were willing to take part in the study and who signed the informed consent were registered as participants. Each participant was registered using the LMS by filling in the required data. After registering on the LMS of the training program, participants were then required to fill out the MCQ before watching a video that contained the materials on the thermography-based foot care for the prevention of DFUs. Each participant had 6 × 24 h to complete the training flexibly and independently. Participants were recorded as having completed 1 video after they had watched 100% of the video. After completing 17 videos, participants were asked to complete the MCQ. This study also surveyed participant satisfaction with the participation in the training by answering a satisfaction questionnaire at the end of the training. Each asynchronous online trainee had a 6 day completion time. This learning program was offered from the 4th to the 16th of December, 2023.

### Statistical analysis

SPSS version 26.0 was used for data analysis. Participants who did not complete 100% of the training by the stipulated period were excluded from the analysis. The MCQ score data were collected from the LMS. In addition to the descriptive statistics, the differences in the participants' scores before and after training were evaluated using paired t tests. Furthermore, interactions were confirmed using the repeated measures analysis of variance. Age was divided into two groups based on the average value and analyzed. All tests were two-sided, with a difference of a *p* < 0.05 value classified as being statistically significant.

## Results

### Study of participants

Of the 150 participants recommended by InWECNA from six islands in Indonesia that were spread across 19 provinces, there were 17 people who did not register for the LMS, and 27 people who did not complete the training for reasons such as being too busy working (*n* = 14), forgetting the training schedule (*n* = 8), poor internet connection (*n* = 4), and difficulty using the internet (*n* = 1). As a result, there were a total of 106 participants who completed the training. The characteristics of the participants are shown in Table 3. The average age of the participants was 40.0 years (SD = 0.9 years). The majority gender was female (57.5%), with participants educated with a bachelor's degree (48.1%), working in hospitals (51.9%), with a majority having > 5 years of working experience. Most participants had a Certified Basic Wound Chronic Nurse (CBWCN) certification with 1–5 years of acquisition.

**Table 3:** Characteristics of participants and interactions in multiple choice questionnaire scores on knowledge of thermography-based diabetic foot ulcer prevention

Variables	N = 106	Questionnaire scores before training	Questionnaire scores after training	p-value (Time)	p-value (Group)	p-value (Interaction)
Age (years) < 39 years > 40 years	40.0 ± 7.9 50 (47.2) 56 (52.8)	51.4 ± 10.9 52.5 ± 9.9	83.5 ± 10.8 86.8 ± 10.2	< 0.001	0.141	0.414
Gender Male Female	45 (42.5) 61 (57.5)	53.0 ± 9.7 51.3 ± 10.8	83.0 ± 11.6 86.9 ± 9.5	< 0.001	0.480	0.046
Education level Diploma Bachelor Master Specialist Doctoral	10 (9.4) 51 (48.1) 29 (27.4) 11 (10.4) 5 (4.7)	50.6 ± 9.5 52.8 ± 10.6 50.5 ± 11.0 50.5 ± 9.8 59.2 ± 4.8	79.8 ± 14.7 86.9 ± 9.2 84.5 ± 10.6 83.8 ± 12.4 87.2 ± 9.2	< 0.001	0.203	0.781
Workplace Hospital Public Health Center Clinic Independent Practice	55 (51.9) 5 (4.7) 10 (9.4) 36 (34)	51.4 ± 9.9 54.8 ± 11.6 56.4 ± 6.3 51.4 ± 11.7	86.8 ± 10.3 87.4 ± 15.2 86.8 ± 8.9 82.2 ± 10.5	< 0.001	0.242	0.433
Work Experience < 1 year 1–5 years > 5 years	7 (6.6) 29 (27.4) 70 (66)	49.1 ± 14.7 50.8 ± 8.8 52.8 ± 10.5	87.1 ± 8.6 84.1 ± 12.8 85.5 ± 9.8	< 0.001	0.614	0.641
Type of Certificate Basic (CBWCN) Intermediate (CWCN) WOC(ET)N	53 (50) 26 (24.5) 27 (25.5)	50.5 ± 10.6 53.9 ± 10.2 53.2 ± 9.9	85.9 ± 10.0 80.2 ± 11.4 88.9 ± 9.4	< 0.001	0.150	0.014
Years Certified < 1 year 1–5 years 5–10 years > 10 years	4 (3.8) 48 (45.3) 33 (31.1) 21 (19.8)	54.0 ± 9.1 50.1 ± 9.5 53.9 ± 11.2 53.1 ± 10.9	93.0 ± 1.15 85.0 ± 11.3 83.2 ± 10.2 87.4 ± 9.8	< 0.001	0.326	0.264
Origin (Island) Java Sumatra Kalimantan Sulawesi Bali Papua	82 (58.5) 16 (15.1) 10 (9.4) 15 (14.2) 2 (1.9) 1 (0.9)	52.4 ± 9.6 48.5 ± 11.1 54.4 ± 8.3 51.3 ± 13.3 65.0 ± 7.1 44	85.8 ± 9.7 84.1 ± 14.1 80.6 ± 8.4 84.9 ± 10.7 95.0 ± 7.1 100	< 0.001	0.261	0.341

*n* (%) or average standard deviation, repeated measures analysis of variance

### Knowledge of thermography-based DFU prevention

Study results showed that asynchronous online training significantly impacted the participants' knowledge. The average MCQ score after training increased by 33.2 points compared to the average MCQ score before training. Before the training, participants had an average MCQ score of 52.0 (SD = 10.3). After the training, the average MCQ score increased to 85.2 (SD = 10.6). The paired sample t test revealed a significant difference between the before and the after training MCQ scores (*p* < 0.001). This suggests there was a substantial improvement in the participants' knowledge due to training.

Table 2 shows the response rate of the wound care specialists before and after training on individual questions on DFU prevention. Out of the 50 items tested, 26 were answered correctly, and 19 were answered incorrectly by more than 50% of the participants before training. After training, all of the tested items were answered correctly by more than 70% of the participants. More than 94% of nurses knew that gait can affect callus formation on the sole. However, over 84% of nurses incorrectly responded that ischemia is not the main factor causing DFUs, and that areas with decreased skin temperature are not an indicator of pre-ulcerative conditions in the legs. After the training, the percentage of correct answers increased on all question items. In fact, more than 90% of the participants answered correctly on question items related to the topics of basic DFU knowledge, self-efficacy, and LOPS standard care and education.

Table 3 presents the results of the repeated measures analysis of variance. Interactions were observed between time (before and after training) and gender, and between time (before and after training) and type of certificate.

### Satisfaction with the training program

Participant satisfaction in asynchronous online training was assessed based on the participant's assessment of the usefulness of the training in improving their understanding of the material regarding the prevention of DFUs based on thermography. The survey results indicated that 89.5% of the participants strongly agreed that the training helped them to understand the training material more deeply, while 10.1% agreed, and 0.4% said they could not definitively decide if it helped.

## Discussion

The results of this study provide valuable insights into the effectiveness of online education for wound care nurse specialists, particularly in the area of thermography-based DFU prevention. The significant increase in knowledge scores from before training to after training demonstrates the potential of using online training programs to enhance clinical competencies among healthcare professionals. These findings align with previous research that has suggested that e-learning can be an effective tool for continuing education in the nursing and other healthcare fields [10, 11]. The e-learning method has been reported to be superior in terms of time effectiveness and cost efficiency [10, 12].

The online training for DFU prevention based on thermography using the Indonesian language was the first program to be released. This training program was very easy for Indonesian wound care specialists to understand as compared to similar training courses in languages other than Indonesian. Asynchronous online training is very appropriate for nurses with a high workload and for those professionals that are geographically working in remote areas, such as archipelagos that are far from the city center, such as wound care specialists in Indonesia. The present training program involved participants from 19 provinces from six islands in Indonesia.

The study highlights several important points. First, the significant improvement in knowledge scores that was observed demonstrates that online education can effectively bridge knowledge gaps in DFU prevention among wound care specialists. Given the critical role of nurses in preventing and managing DFUs, this finding has important implications for patient outcomes. Enhanced nurse education can lead to better patient care practices, thereby ultimately reducing the incidence and recurrence of DFUs and improving the quality of life of patients. Although this training was delivered online, the advantage of the training in this study was the use of videos containing basic concepts of DFU prevention and step-by-step demonstrations of the preparation, process, and evaluation of DFU prevention procedure techniques using thermography as well as case studies of the application of the procedure. Thus, participants are expected to gain a deep understanding and be motivated to demonstrate the DFU prevention protocol using thermography directly.

Second, the attrition rate observed in this study (with 27 out of 133 registered participants not completing the training) points to the challenges that need to be addressed to maximize the effectiveness of online education. However, the proportion of participants who dropped out in this study was still lower (20.3%) than that reported in a previous survey, which reached 30% [13]. Factors such as a heavy workload, forgetfulness, poor internet connectivity, and difficulty using online platforms were cited as being reasons for non-completion. These barriers highlight the need for flexible and user-friendly online training programs and support mechanisms to help nurses balance their training with their professional responsibilities.

Third, the present findings on the relationship between the participant characteristics (gender and type of training certificate) and the increase in knowledge are noteworthy. This relationship suggests that a tailored approach to education may be more beneficial, although it is difficult to examine the background behind the interaction based on these data. For example, additional support or training formats based on participant characteristics may be required to ensure fair and effective learning outcomes across different groups.

Although this study found that online training can improve the knowledge score of wound nurse specialists, we need to point out that there were participants who had low knowledge scores even after attending these training programs. Various factors can affect the low score results, including participant factors, training/program factors, and environmental factors [14]. Participant self-motivation is a psychological issue that can reduce participants' interest in participating in training. In addition, the use of an interesting and creative training program can increase the enthusiasm and performance of participants [15]. Other factors include environmental support in the form of work commitments, various family and social responsibilities, and inadequate support from family, friends, or co-workers. Thus, increasing motivation, developing more interactive training and environmental support can increase the enthusiasm and focus of the participants who participate in the training. However, these results do show that there is an increase in the knowledge score after participants undergo training.

Overall, this research contributes to a growing body of evidence supporting online education in the healthcare field. It underscores the importance of continuing education for nurses and provides a model for implementing effective online training programs. Future research will need to explore broader outcomes and investigate the long-term impact of such training on clinical practice and the benefits for patients. Additionally, examining the specific needs and preferences of different subgroups of health professionals can help in the design of more effective and inclusive educational interventions.

## Conclusions

The results of this study demonstrate that asynchronous e-learning significantly enhanced nurses' knowledge regarding the prevention of DFUs using thermography. After completing the online training, the quasi-experimental design revealed there was a significant improvement in knowledge scores among the wound care specialists. Despite some participants not completing the training for various reasons such as workload and technical issues, this training can be held in large numbers with an even distribution of participants. The study also identified that gender and the type of training certificate held by participants exhibited significant relationships with the improvements observed in their subsequent knowledge scores. These findings suggest that online educational interventions are effective and can be tailored to meet the needs of healthcare professionals, thereby ultimately contributing to better patient care outcomes in preventing DFUs.
